# The Asylums of Italy, Germany, and France

**Published:** 1858-01-01

**Authors:** John T. Arlidge

**Affiliations:** Member of the Royal College of Physicians of London; formerly Resident Medical Officer of St. Luke's Hospital, London


### Art. III.—THE ASYLUMS OF ITALY, GERMANY, AND FRANCE.

### NOTES OF A VISIT MADE IN THE YEAR 1855.

BY JOHN T. ARLIDGE, M.B., A.B. (LOND.),

Member of the Royal College of Physicians of London; formerly Resident Medical Officer of St. Luke's Hospital, London.

(*Continued from* Vol. x. page 775.)

*Venice* possesses two public asylums—viz., one for males, occupying the small islet of St. Servilio, a short distance from the city; and the other for females, situated at one extremity of the town, and forming a department of the large civil hospital.

wicket was open, and through it the enemy kept up a heavy fire upon them. Sergeant Carmichael was killed while laying his powder-bag, Havildar Mahor being at the same time wounded. The powder being laid, the advanced party slipped down into the ditch to allow the firing party, under Lieutenant Salkeld, to perform its duty. While endeavouring to fire the charge, Lieutenant Salkeld was shot through the arm and leg, and handed over the slow match to Corporal Burgess, who fell mortally wounded just as he had successfully accomplished the onerous duty. Havildar Tilluh Sing, of the Sikhs, was wounded, and Ramloll, Sepoy of the same corps, was killed during this part of the operation. The demolition being most successful, Lieutenant Home, happily not wounded, caused the bugler to sound the regimental call of the 52nd, as the signal for the advancing columns. Fearing that amid the noise of the assault the sounds might not be heard, he had the call repeated three times, when the troops advanced and carried the gateway with complete success. I feel certain that a simple statement of this devoted and glorious deed will suffice to stamp it as one of the noblest on record in military history. The perfect success contributed most materially to the brilliant result of the day, and Lieutenants Home and Salkeld, with their gallant subordinate Europeans and natives, will, I doubt not, receive the rewards which valour before the enemy so distinguished as theirs has entitled them to."

The origin of both of these institutions or the insane is comparatively very recent. No State provision was made for the insane poor until 1802, when Ludovico Manin, the last Doge of Venice, gave by will a sum of 55,000 ducats for their maintenance, under the direction of the brethren (Padri Ospitalieri) of St. John the Divine (S. Giovanni di Dio), popularly known in Italy as the "Fate-bene-Fratelli." Prior, indeed, to that date—from the year 1737—a few patients belonging to the upper classes of citizens had been committed to the care of the brethren, at the expense of their families, and in 1797 had reached 30 in number. But the charge of such patients remained only subordinate to the general purpose of the institution as a military hospital; and it was not till after Manin's munificent bequest was received that the care and treatment of those mentally disordered became a primary object with the benevolent monks, and that an asylum for the insane of Venice rose into existence.

In 1822, the increasing number of lunatics in the monastic institution at St. Servilio induced the authorities to seek further accommodation elsewhere; when the ancient hospice of the mendicants connected with the great civil hospital of St. John and St. Paul, within the city, was selected, and, by various alterations and additions, fitted for their reception.

Soon after the institution of this second asylum, it was judged expedient to retain refractory and noisy cases at St. Servilio, and to transfer the quiet, convalescent, and the imbecile to the establishment in the city. As yet the monks had admitted both male and female patients under their care; however, in 1834, it was resolved to separate the sexes, and to locate the males in St. Servilio, and the females in the city asylum. This scheme was carried out in the following year, and is still adhered to. In each asylum two classes are received—viz., paupers and pensioners.

We shall commence with a description of the asylum for the female sex, of which we have, thanks to the kindness and courtesy of M. Pelt, the able chief physician, ample details both of its structure and management.

The Venetian asylum for female lunatics is situated at one extremity of Venice, bordered on the north side by the Lagoon; on the west, by the wide canal "de' Mendicanti;" on the south, by the General Hospital and the Chapel; and on the east, by a narrow street of adjoining houses. In position, therefore, it is much confined; and the only prospect from it is on the north side, extending over the Lagoon to the mainland and its distant mountain chain.

As before intimated, the building was originally destined for an asylum for aged and infirm mendicants, and has only of late years been converted to its present purpose. According to the prevailing system of construction of Italian public institutions, it is so built as to enclose a square court, with a covered arcade running round it: and is of three stories. The basement is occupied by the offices of the physician; the visiting-room; the work-room; the refectory or dining-room; the "spinning" or weaving-room; a dormitory for epileptics, one for melancholic, and one for demented patients; by the bath-room, the kitchen of the infirmary, and store-rooms. On the first floor are—the large dormitory, partially subdivided into three rooms for maniacal patients; the dormitory for monomaniacs, the infirmary, and the apartments set apart for the class of pensioners, and reached by a distinct staircase. On the floor above are other dormitories—one for the reception and observation of new cases prior to their distribution, and another for the most refractory cases.

The central court-yard is the only available space for exercise, except a garden situated at some distance from the asylum, and therefore little available for general or frequent use. In the centre of the court is a fountain of good water. The court was paved, but not in a satisfactory state. The surrounding corridor, supported upon columns, had a pleasing appearance, and afforded a welcome shady walk in hot weather. It is beautifully paved with a sort of concrete, common in the country, composed of fragments of marble rolled and fixed into some kind of cement, which becomes very hard, and has a fine, smooth surface. The floor of this arcade is unfortunately—so far as concerns feeble patients—elevated between 1 foot and 18 inches above the level of the central court, and thus forms an awkward and dangerous step. The roof of the arcade constituted a promenade to the first floor—little available, however, to the inmates, from being too dangerous.

The work-room, which also serves at other times as an amusement-room, is of considerable, although of insufficient size for the number of patients daily resorting to it. It is bare of ornament, and dull from the height of the windows above the ground, and from their being closed by Venetian shutters, reaching two feet from the bottom. Adjoining it is the "spinning-room" (Filanda), so called because the patients are occupied in it at spinning-wheels and small looms, in preparing the flax, &c. The class so employed consists of those inexpert in needle-work, or who are unfit to mingle with the cleaner and more orderly inmates of the common work-room.

These rooms, as well as the dormitories, have the same sort of concrete floor as the arcade; but M. Pelt has, in some of them, had a wooden floor laid above it, as it was found to be cold and injurious to many weak patients. The rooms on the basement are about 13 feet high, and are lighted with gas at night. The work-rooms have also a central stove, protected by a stout wooden railing. The walls are whitewashed and bare; the furniture consists of plain wooden tables (not fixed), chairs, and settees. The "spinning-room" is entered from the corridor by a very prison-like barred iron door, kept always locked: like the other rooms on this floor, it is elevated by two or three steps above the corridor, a constructional peculiarity open to many objections.

The baths are constructed of marble, and can be supplied with hot and cold, salt and fresh water. A douche-pipe is attached above each of them, and by screwing a rose on the end of it, a sort of shower-bath is obtainable.

In the small visiting-room, on the ground floor, was a piano for the use of the patients. This room conveniently communicated on one side with the large work-room.

The dormitories are intended only for night use; when patients require from any cause to keep their bed, they are transferred to the infirmary, a large and lofty apartment on the first floor, containing 28 beds, arranged along the walls on either side, having the usual concrete floor, and a large stove in its centre. The windows are placed some five feet above the floor—a very faulty arrangement, whether the ventilation or the cheerfulness of the apartment be considered. To each bed in this infirmary are allotted a "table de nuit," and a shelf for books.

The dormitories hold from 20 to 30 beds each, and contain no other furniture. A crucifix or a Madonna is commonly affixed against some part of the wall; and a gas-light, suspended from the centre of the room, serves to illuminate it at night. There is a special provision made to secure efficient ventilation, by the formation of apertures just above the floor, of about a foot square each, placed at considerable intervals, and communicating with the external air. These are closed on the inside by swinging doors or shutters, and externally by stout wire-work.

On this first floor was a room for seclusion, of about 14 feet by 15 feet, with a window placed high up, which could be closed by a shutter, when it was desirable to darken the room.

The more refractory patients sleep together in a dormitory on the second floor—the majority of them being under some sort of restraint during the night. A few indeed are retained in it during the day confined to their beds.

The portion of the asylum set apart for the pensioners (who are of two classes) consists of a common dining or day-room, of two dormitories, having six beds each for those of the second-class, and of four single rooms for first-class inmates, besides a chamber furnished with a restraint bed. Every first-class patient has her own attendant, who sleeps in the same apartment with her. These single rooms, and likewise the general day-room, are comfortably furnished.

The bedsteads for the indigent patients are of very cheap construction, consisting of an iron framework with a headpiece, supported on a pair of iron trestles at the head and foot. The bedding consists of a paillasse stuffed with straw or shavings, of a flock bed, an upper and under sheet, a coloured woollen coverlet, and over this a printed and coloured cotton one. Some of the coverlets were quilted, to give them greater strength; the sheeting was also of coarser or finer material, according to the exigencies of the cases.

In northern Italy the bed and paillasse constitute, among the poor, the marriage portion of the bride, and are transmitted from generation to generation, the possession of several such heirlooms being a matter of pride. Hence it is common to see one or two beds or paillasses piled one on the other, giving the happy possessor an elevated resting-place, but one, by the way, from which it would be disagreeable to fall. This partiality for high beds persists in the asylums, and is indulged to a certain extent; some patients being allowed a very thick paillasse, or two of them, beneath the thick bed. The bedsteads themselves are actually low; yet by the quantity of bedding laid on them, the patient would appear raised to a dangerous height above the ground. However, I was assured that no accident had ever occurred from this custom; and we must conclude that, by habit, their movements asleep are so circumspect that they risk no fall from their high estate, but poise themselves securely, just as our German friends manage, by their much-to-be-admired quietude in bed, to balance and retain the light upper bed upon them, and nestle under its warmth, whilst our unskilled countrymen speedily part company with it, and tire in the attempt to ensconce themselves beneath it.

The luxury of a high bed, as may be supposed, is denied the epileptics; yet no specially constructed bedsteads are in use for them. Paralytics are occasionally furnished with feather-, in place of the common wool-beds. Water-beds and water-cushions are unknown, and even air-cushions seem never to have been introduced into use in any of the Italian asylums. For dirty cases, a sort of trough or case is fitted into an iron framework, and filled with straw; over this a piece of stout linen or canvas is extended, upon which the patient is placed. The urine percolates through the straw, and is collected in a clumsy wooden tray beneath.

The laundry and the dispensary are common to the asylum and to the general hospital, of which the former is only a section. The laundry gives employment to a considerable number of patients; but by its distance from their own section, and by its appertaining equally to the general hospital, they are to a certain and injurious extent removed from the special supervision and control required; an evil further augmented by their being under the direction of servants unconnected with the asylum. Another objection to the laundry was the employment there of two or three men, who mixed among the female patients.

Like those just named, the other domestic offices, such as the kitchen for the cooking of food, the clothing and victualling departments, were common both to the asylum and hospital, and under the jurisdiction of the officers of the latter.

The staircases are straight, but some of them open on one side, and are so far dangerous. The windows are everywhere protected; those on the ground-floor by bars externally, and by wire-work within; and those on the upper floors by the wirework alone.

*Internal Organization and Treatment.*—As the asylum forms a part of the Civil General Hospital, it is placed under the supreme control of the director-general of the whole establishment. Nothing can be undertaken, and no expenses incurred, without his assent; and he is supposed to supervise the entire management and internal economy of the institution. The appointment of chief physician and of the assistants to the asylum is vested in him. In the economical details he is assisted by the steward (administrator). The moral discipline and medical treatment are left to the chief physician, who is responsible for their due execution to the director-general, but not for the particular medical treatment he may adopt in any case. He is non-resident, and makes two visits daily—the principal one at 8 A.M. He has under him two assistants, or "internes," who reside in the asylum, receive their board, and one of them a very small salary. They are appointed for two years, and, if recommended, may be re-appointed for two more; they are, however, not occupied throughout the whole period in the asylum, but from time to time act as "internes" in the general or other hospitals, according to the excellent system prevailing in France. They are required to attend the physician during his visits, to write out the tables of diet, to supervise the distribution of the medicines, to bleed and to perform other minor surgical as well as medical duties which may arise in the asylum, whether by night or day; consequently they are not allowed to absent themselves from the building, except by permission of the physician or of the director.

Next in order is the head attendant, who has two deputies beneath her; and is, with them, under the direction of the physician as to all medical and moral matters affecting the patients, and also responsible to the steward for the clothing, furniture, &c., under their charge. "A good head-attendant (Dr. Pelt well remarks) is not only equivalent to the continued presence of the physician, but also a great aid to him in his duties; for he lives among the patients, is domesticated with them, and has it in his power to note their failings, and to arouse the latent energies of their torpid intellects."

The number of nurses varies according to the demand for their services; usually there are about thirty. Their relative number is greatest in the refractory ward, and next to this in the infirmary. In the other sections, containing more orderly patients, the attendants are in the proportion of one to fifteen. The paying patients of the first class have each an attendant, and it is chiefly due to the varying number of such inmates that the total of the staff of nurses fluctuates so considerably from time to time. The remuneration of the attendants increases with their length of service. Those who are married are allowed to go out one day in every five; the unmarried, once in fifteen days; the night duty is taken by them in turns.

Besides the ordinary attendants, there are five others to instruct and to superintend the employments of the patients. One is chief, and has the especial charge of the great work-room. Unlike the nurses, they are hired to attend only during the hours of labour—viz., for four before, and three after dinner.

At the time of my visit, 260 patients were under treatment: a number almost as great as the institution could accommodate. A scheme has been talked of, to extend the present building, by purchasing the houses in the narrow street bounding it on the only side where enlargement is possible; but this, I trust, will never be carried out, for it would tend to perpetuate the existence of the asylum in a most unfit locality, and under the trammels of the general hospital.

The asylum receives patients from the entire province of Venice,—from Vicenza, Padua, Verona, Polesino, Treviso, Feltre, Belluno, and Friuli. Some are also sent from Dalmatia; and, until the asylum of Halle, in the Tyrol, was instituted, patients were likewise forwarded from that province.

A system prevails of collecting the insane in the general hospitals of the larger cities, from which they are sent on in batches to Venice .The misfortune and evil of this plan is, that, just as happens among ourselves in our workhouse infirmaries, the patients are placed under unfavourable circumstances, are often maltreated or neglected, and their disease suffered to grow worse, and too often beyond the prospect of cure. This evil, M. Pelt has most sensibly pointed out in his printed reports.

The entirely dependent, or pauper class of patients, when attacked with acute or insidious mania, are supported from the imperial treasury; but when harmless—not dangerous—incurables, when idiotic from birth, or when pellagrous, they are maintained at the cost of the commune in which they were born, or of that in which they have been domiciled for the last ten years. It is left for the commune to recover the cost from the relatives, on whom it should rightly fall, where this course is practicable.

The charge to those who have just sufficient for their maintenance is their actual cost to the institution—viz., about tenpence per day. These fare with the indigent; but those who are able to pay a shilling a day constitute the second-class of pensioners, and live, as before noted, in a distinct portion of the institution, and have a superior table. Lastly, the first-class patients pay at the rate of half-a-crown a day, have the same table as the second grade, and individually a separate room and attendant.

Pensioners are admitted into an asylum on producing a medical certificate, viséd by the commissary of police, and accompanied by a petition setting forth the class to which they wish to be attached, by a month's payment in advance, and by a guarantee to continue the payment during their residence in the establishment.

The affair is much more complicated when a pauper case seeks admission. A paper has to be filled up, exhibiting a parish certificate as to his or her poverty; a statement by the medical man in previous attendance; the assent of the communal authorities to undertake all the necessary cost; the opinion of the physician of the province, respecting the stage and form of the malady, except in the case of Venice; a certificate of age, of parentage, place of birth, residence, occupation, civil position, date of attack, the history of predisposition, of the acts of insanity committed, of the course of the treatment pursued. One copy of this document is retained by the police authorities, and another by those of the hospital.

In Venice, much power is lodged in the hands of the medico-fiscal of police, who can amend the returns made by the provincial physicians, and transfer patients to or from the charge of the communes, according as he judges them to belong to the category of acute cases, or to that of the harmless and chronic.

On the entrance of patients into the asylum, they are stripped of their clothes, washed, and then clothed in the common uniform of the institution. Their old clothes, after being cleansed, are laid aside, and entered in an inventory. Each patient's name is entered in a register, in which all the particulars gathered from the certificates supplied, and all details obtainable respecting the past history and habits, are recorded. A daily account is also kept, by the physician and assistants, of the diseased manifestations both of mind and body, of their modifications, of the treatment, the employment and diet prescribed, and of any other particulars required to fill up the medical history of the case.

When any intercurrent disease arises, and the patient is transferred to the infirmary, the case is entered on the distinct and special register of that section of the asylum. The advantage of this proceeding is very doubtful.

A commission meets every month, composed of the physician of the province, of the fiscal and one commissary of police, of the police registrar, and of the chief physician of the asylum, who submits his reports of the cases. This commission determines what patients are to be discharged cured; which may be entrusted to their friends, owing to their improved state; and which have become harmless, and may be transferred to some other institution.

Five varieties of diet are in use: Broth (bouillon) alone, as low diet.

I. A soup containing one ounce of vermicelli at dinner, and a like quantity of bread for supper.

II. A soup having in it an ounce and a half of vermicelli, an ounce of bread, and one of boiled veal, at noon; the supper as above.

III. A cup of coffee and one ounce of bread in the morning; for dinner, once in the week, a soup containing three ounces of rice, or two of barley; on other days, two ounces of boiled beef, two of bread, and four of wine; for supper, an equal allowance of bread, boiled in the broth. This description of diet is allowed the pensioners when in health, but it is cooked separately, and better seasoned; and at breakfast they have coffee with milk, and a sort of fancy bread (ciambella).

IV. The fourth description of diet includes an ounce more of each article contained in the last, besides the quarter of a fowl or two ounces of roast veal, or else of fried liver with cream, one or the other alternately. In the evening, besides three ounces of bread, either an egg, or some cheese, or legumes, with a smaller allowance of wine than at dinner, is allowed.

V. The common diet of the majority consists alternately of two ounces of bread and six of porridge on one morning; of eight of porridge and half an ounce of cheese on another; four ounces of rice or three of barley, two ounces of boiled veal, four of bread, and six of wine, for dinner; four ounces of bread, four of wine, with half an ounce of cheese, of legumes, or of salad, for supper.

It is in the power of the physician to vary the diet in any manner he sees fit, and to allow extra articles. The diet table is made out every day, according to his directions, by his assistants. The women employed in the laundry are always allowed, in addition to the ordinary dietary, a pound of porridge and half an ounce of cheese in the course of each day.

The breakfast is given at half-past eight; the dinner at half-past twelve; and the supper at seven in the evening. The meals are eaten together in the common dining-room. The patients are kept at their several employments from nine till twelve, and from three till six; and thus sufficient time is afforded them, in the middle of the day, for rest or recreation; the misfortune is, that the institution affords no better scope for the last than the limited central court, and its surrounding arcade. At night, in the winter, the common rooms are lighted with gas, and, from time to time, dancing goes forward, or music or marionnettes chase away the monotony and dreariness of the time.

Morning prayer is not forgotten, for those who can participate in it; and on feast days the majority attend mass in the chapel, and afterwards receive visits from their relatives. On such days, too, the patients are treated, after dinner, to fruit or cakes; and if the weather be bad, amuse themselves indoors with dancing and singing, or attend the musical services in the chapel—sacred music, and the organ, almost invariably affording delight. When the weather is good, a select number are allowed to walk in the garden belonging to the hospital.

The visits of friends are restricted to the two hours between ten and twelve o'clock, except those to pensioners, or where the relatives come a considerable distance, when a degree of indulgence is granted. The pensioners receive their friends in their own apartments; the rest of the inmates see theirs in the common visiting-room.

Uniformity of dress is the rule of the asylum; but it is slightly infringed by the grant of finer or more ornamental articles of clothing, given by way of encouragement and of reward, chiefly to those profitably employed in the institution. For the work done by the inmates, a regular valuation is made by the steward, and a certain proportion of the estimated value is set aside for their use and gratification. In winter, the outer dress is woollen; in summer, of striped cotton. I observed a few of the women disorderly in dress, but in general their condition was very satisfactory. The plan of strengthening the dress, or of otherwise modifying its form to meet the wants of special cases, did not appear to have occurred to the medical directors. In the instance of the shoes, we must, however, admit an exception to this statement; for in order to retain them on the feet, in certain cases, a leathern strap was crossed over the instep and fastened by a screw in the thickened sole, beneath the hollow of the foot.

The head nurses were very neatly dressed, but some of the inferior attendants were less tidy than could be wished. A particular style of dress is assigned for the use of all the attendants.

Mechanical coercion is much resorted to in case of violence, of destructiveness, and of suicide. In certain cases it is deemed indispensable; those are particularly cited in which the patient attempts to inflict injury without exhibiting marked *furor*, but, as it were, maliciously and insidiously.

The means employed are—the strait-waistcoat, made with long sleeves, which are fastened behind, the arms being crossed in front; leathern straps, used to confine the hands, the body, or the feet, in bed; a restraint bed, constructed as a long wooden box, which is filled with straw, and covered in by a strong open trellis-work—in this, the patient is fastened by the feet to the end of the "bed" by means of straps, sometimes also by the hands, and in extreme cases by a broad canvas band stretched across the body.

This terrible contrivance, which as little deserves the name of "bed" as any machine devised for torture and to chase away repose, has, in order to meet the requirements of the pensioners, and to adapt it to their more refined notions of elegance and ease, been constructed of a "sufficiently elegant pattern, in iron," and has its edges padded.

Again, patients are confined in the ordinary beds by one or both hands or feet. Thick leathern gloves are also used at night, chiefly in cases of self-abuse. Handcuffs made of two leathern rings, connected by one or two iron links; a strap round the waist, to which the hand on each side is fastened by another strap, are among other instruments of restraint still in use, and conscientiously believed to be imperatively necessary to the welfare of the patients and the good conduct of the establishment. The feet are rarely fastened together by day; but in the refractory dormitory I saw one woman confined by one leg, by means of a ring and strap, to a fixed seat.

At the time of my visit I did not count more than eight under restraint; at night this number would be greatly increased.

Seclusion in a darkened room is reckoned among the means of repression or restraint. "The sudden forced suspension of the senses," says M. Pelt, "acts at times upon the mind with advantage, and frequently allays *furor* in a few minutes. In some cases, however, it augments the incipient cerebral congestion, and is a resource not to be indifferently employed." The plan of secluding patients he disapproves of, and prefers restraint to it.

In the medical treatment of the insane, M. Pelt is guided by the nature of the concomitant disorder—whether this affects the digestive or the sanguiferous system. He insists much upon derangements of the liver and digestive canal as concomitants or consequences of the mental disorder, and considers their relief to be the primary object of treatment. To relieve disorders of the circulating system, he employs local bleeding by leeches or cupping, blisters, issues, setons, the douche, the application of ice to the head, and baths. Venesection is, in his opinion, very seldom required, although it may be called for in threatened congestion, or when a patient is very robust, or has contracted the habit of losing blood at intervals. He also very justly remarks that the excitement of mania induces and is followed by prostration, which can only be increased by the withdrawal of blood; and that the less elastic or depressing character of the air at Venice is of itself a reason against so debilitating a mode of treatment. In lieu of it he prefers to apply leeches to the anus, or, in cases of nymphiomania, to the nape of the neck. Blisters are of limited applicability, on account of the generally hot and dry state of the skin, when they cause greater irritation than they can do good. The actual cautery, although cruel in appearance, may be used without misgivings on account of the ordinary insensibility of the insane, and is of great advantage in active mania, dependent on the exacerbations of chronic meningitis. M. Pelt showed me a case of epilepsy complicated with chorea, in which the paroxysms were very frequent, where he applied the actual cautery on each side of the spine, for the length of eighteen inches, with very marked benefit.

The douche is commonly given when the patient is in a warm bath; it is allowed to fall either in a strong stream, or by drops, or through a rose, in a fine shower, upon the head, and is principally used in cases of mania and monomania. Its application in force for a few minutes is sufficient; if continued longer, it produces fainting. The bore of the pipe is varied in size according to circumstances; but the use of a large stream is rare, except as a means of repression. Warm-baths are employed medically and frequently, but not regularly or systematically, for the purpose of cleanliness. They are always abstained from in cases of paralysis, apoplexy, and epilepsy. Ice in bladders to the head is principally useful where there is actual cerebral inflammation.

Tartar emetic is much used to allay excitement. Opium is rarely given, because it is supposed to produce hallucinations, to fail in ensuring healthy sleep, and to be further objectionable by its stimulating properties.

The moral management of the patients is carried out by insisting on kindness and patience of manner towards them on the part of their attendants and others; by encouraging them to employ themselves; by returning to them a portion of the proceeds of their labour, &c.; by providing them with all possible recreation, and with amusements. No coercion is used to induce them to work. Reading is almost entirely limited to the pensioners, most of the common patients being unable to read. An attempt was made to give them "readings," but it failed, chiefly from not sufficiently arousing their attention, and from the drowsy inclinations of the majority.

In the female population of this asylum, 20 of the 260 were epileptic at the time of my visit; but M. Pelt states 15 to be about the average number. General paralysis is very rare—at least, it is so stated; but it appears that many of the Italian physicians are but indifferently acquainted with its true character. The statement of M. Pelt, that he has cured cases by nervine remedies, epispastics, and frictions, must, I think, be received *cum grano salis*; for a doubt arises whether they were genuine examples of the disease.

Before quoting the statistics, a few miscellaneous particulars are worth noting.

The following classification is adopted in the Venice Asylum:—

Class I.—*Mania.*

1. Sub-class . . . . . *Monomania.*
2. " . . . . . . *Melancholia.*

Class II.—*Dementia.*

1. Sub-class . . . . . . Acute.
2. " . . . . . Chronic.

Appended division . . . . . . . . . Idiocy.

A peculiar plan was started in this asylum a few years ago, by Dr. Fassetta, of indicating the class or sub-class of mental disorder by a particular colour—viz., mania, by red; monomania, by blue; melancholia, by green; acute dementia, by yellow; chronic dementia, by a different shade of (pinkish) yellow; and idiocy, by olive. With the notion of promoting order and method in the establishment, these distinctive colours are woven in the dress of the patients, so that the particular form of their malady is at once patent to the eyes of the initiated observer.

The asylum is inspected every month by the Physician of the Department, and the Commissary of Police; and a monthly report is required by the Government, in addition to one returned every fortnight to the local authorities.

An excellent regulation enforces six months' attendance at this asylum of all candidates for a degree in medicine. We may well take shame to ourselves, that in this country our governing bodies have not yet seen the necessity for such a regulation; but, on the contrary, have ignored the study of psychological medicine, whilst they have made natural history an integral part of the medical curriculum.

TABLE I.

*Movement of the Population in* 1846, *according to the form of Insanity.*

| Form. | Existing. | Admitted. | Discharged. | Dead. | Remaining. |
|---|---|---|---|---|---|
| Mania | 86 | 102 | 62 | 29 | 97 |
| Monomania | 34 | 25 | 17 | 6 | 36 |
| Melancholia | 34 | 24 | 23 | 12 | 23 |
| Acute dementia | 41 | 36 | 10 | 28 | 39 |
| Chronic dementia | 65 | 8 | 1 | 19 | 53 |
| Idiocy | 12 | 1 | 1 | 5 | 7 |
| Total | 272 | 196 | 114 | 99 | 255 |

The mortality this year was rendered so considerable by the prevalence of pellagra among those newly admitted. In 1844 and 1845, the total in figures stood thus in each year:—

| | Existing. | Admitted. | Discharged. | Dead. | Remaining. |
|---|---|---|---|---|---|
| 1844 | 268 | 192 | 77 | 115 | 268 |
| 1845 | 268 | 189 | 113 | 72 | 272 |

In 1844 the high range of death was due to the same cause as in 1846, which in 1845 had been in a great measure overcome. Still, even in this, the most favourable year as far as concerns the mortality, this last is exceedingly great when compared with that in our own asylums, although explicable by the havoc caused by pellagra, as subsequent Tables will presently show. The total number in that year under treatment being 457, and the deaths 72, above one-sixth (6·347) died—*i. e.*, about $17\frac{6}{7}$ per cent. The year 1844 was exceptional, on account of certain changes going forward in the organization of the asylum: setting it aside, therefore, and taking 1845 and 1846, we find that of the total number under treatment, nearly one-fourth was discharged, or 25 per cent. No division of the number discharged, into cured and relieved, is attempted; however, M. Pelt says the proportion of the latter does not exceed 6 per cent., although it includes incurables, who are sent away to some other establishment, or to their friends, as harmless. Deducting this 6 per cent. from the 25 per cent. discharged, it would appear that the ratio of cures to the total number under treatment in the year is about 19 per cent.

TABLE II.

*Relative Ages of Patients.*

| | Existing. | Admitted. | Discharged. | Dead. | Remaining. |
|---|---|---|---|---|---|
| Under 10 years | 2 | 3 | — | 3 | 2 |
| From 10 to 20 | 13 | 16 | 10 | 14 | 5 |
| " 20 " 30 | 68 | 119 | 68 | 58 | 61 |
| " 30 " 40 | 71 | 165 | 90 | 60 | 86 |
| " 40 " 50 | 72 | 119 | 68 | 61 | 62 |
| " 50 " 60 | 32 | 86 | 45 | 47 | 26 |
| " 60 " 70 | 8 | 48 | 18 | 27 | 11 |
| " 70 " 80 | 1 | 16 | 5 | 10 | 2 |
| " 80 " 90 | 1 | 5 | — | 6 | — |
| Total | 268 | 577 | 304 | 286 | 255 |

This Table exhibits the great proclivity of females to insanity during the period of greatest uterine activity. The maximum is reached between the 30th and the 40th year—the fourth decennial period.

TABLE III.

*Civil Condition of the Patients.*

| | Existing. | Admitted. | Discharged. | Dead. | Remaining. |
|---|---|---|---|---|---|
| Married | 122 | 260 | 136 | 149 | 97 |
| Widows | 36 | 110 | 59 | 49 | 38 |
| Unmarried | 110 | 207 | 109 | 88 | 120 |
| Total | 268 | 577 | 304 | 286 | 255 |

Hence it is shown that the married are most frequently the subjects of insanity. The value of this fact would be more precise, did we know the civil condition of the whole adult population of Lombardy, and the relative proportion of married and single women.

TABLE IV.

*Causes of the Malady.*

| | Existing. | Admitted. | Discharged. | Dead. | Remaining. |
|---|---|---|---|---|---|
| Moral, generally | 112 | 153 | 93 | 72 | 100 |
| Physical, generally | 61 | 80 | 36 | 60 | 45 |
| Luxury and pride | 16 | 17 | 13 | 8 | 12 |
| Excesses | 4 | 34 | 15 | 7 | 16 |
| Pellagra | 42 | 248 | 130 | 103 | 57 |
| Epilepsy | 11 | 18 | 4 | 11 | 14 |
| Old age | 2 | 22 | 3 | 19 | 2 |
| Unknown | 20 | 5 | 10 | 6 | 9 |
| Total | 268 | 577 | 304 | 286 | 255 |

This Table brings to our notice one of the greatest modern scourges of Lombardy—viz., Pellagra, which we find tabulated as the cause of insanity in 290 cases of 845 under treatment; as fatal in above two-fifths of those admitted suffering from it, or in more than one-third of the total number of cases; and as the cause of 103 out of the total of 286 deaths.

The admissions are most numerous in summer, particularly in June and July. The deaths, on the other hand, augment between July and November; but the difference in their relative frequency in the several months is not nearly so striking as that of the admissions.

TABLE V.

*Immediate Causes of Death.*

| DISEASES. | Mania. | Monomania. | Melancholia. | Acute Dementia. | Chronic Dementia. | Idiocy. | Total. |
|---|---|---|---|---|---|---|---|
| Meningitis . . . . . | 2 | — | — | 1 | — | — | 3 |
| Bronchitis . . . . . | 2 | 1 | — | 3 | — | — | 6 |
| Pleuritis . . . . . . . | — | — | — | — | — | 1 | 1 |
| Gastritis . . . . . . . | 4 | — | — | 1 | 1 | — | 6 |
| Hepatitis . . . . . . . | 1 | — | — | — | — | — | 1 |
| Enteritis . . . . . . . | 5 | 1 | 2 | 2 | — | — | 10 |
| Arthritis . . . . . . . | — | — | 1 | 1 | — | — | 2 |
| Phthisis . . . . . . . | 6 | — | 2 | 2 | 1 | — | 11 |
| Marasmus . . . . . . . | 10 | 4 | 14 | 18 | 11 | 1 | 58 |
| Ascites . . . . . . . | 3 | 3 | 1 | 1 | — | — | 8 |
| Pulmonary catarrh . . . | 4 | 1 | 3 | — | 3 | 1 | 12 |
| Precordial disease . . . | — | 1 | 1 | 1 | 1 | — | 4 |
| Apoplexy . . . . . . . | 3 | — | 1 | 4 | 3 | — | 11 |
| Paralysis . . . . . . . | 3 | — | — | 7 | 7 | 1 | 18 |
| Epilepsy . . . . . . . | 3 | 2 | 1 | — | 3 | 1 | 10 |
| Diarrhœa . . . . . . . | 28 | 2 | 12 | 15 | 21 | 3 | 81 |
| Dysentery . . . . . . | — | 1 | — | — | — | — | 1 |
| Scurvy . . . . . . . | 6 | 3 | 8 | 7 | 4 | 1 | 29 |
| Cerebral congestions . . | 1 | — | — | 1 | — | — | 2 |
| Erysipelas . . . . . . | — | — | — | — | 1 | — | 1 |
| Articular abscess . . . | 1 | 1 | — | — | 1 | — | 3 |
| Variola . . . . . . . | 1 | — | — | 1 | 1 | — | 3 |
| Old age . . . . . . . | — | — | — | — | 5 | — | 5 |
| | 83 | 20 | 46 | 65 | 63 | 9 | 286 |

Diarrhœa takes the lead among the immediate causes of death—a fact at once explicable by that disorder being the most frequent complication of pellagra. It occurs either in a colliquative or ulcerative form. The next most prevalent cause stated is, marasmus; but, unfortunately, this is usually only a symptom of some internal or constitutional disorder. That it figures so largely in the above Table is doubtless due to its being a very frequent consequence of the most widely-acting cause of insanity in this region—viz., pellagra. Scurvy stands third in order among the causes of death, and is due, like pellagra itself, to the insufficient nutriment obtained by the poorer classes, which mainly consists of Indian-corn. The relative proportion of the other assigned causes in the Table calls for no particular observation.

From a review of the history of this Venice Asylum, the reader will, we think, be ready to admit that much merit is due to its managers for its internal organization and superintendence. They have to contend against the greatest disadvantages of site, the very indifferent adaptation of its structure to the wants of lunatics, and the daily difficulties involved in its connexion with the general hospital. It is sad to have to record the employment of so much mechanical restraint; but we must allow for the prevailing prejudices of the physicians of the country in its favour, and for the impediments to its entire abolition existing in its confined site and inappropriate structure: and we may hope that the diffusion of the knowledge of what has been and is done in British Asylums will encourage our Italian coadjutors in psychological medicine to imitate our practice. On the contrary, it is highly gratifying to see how much has been done in Italy for the helpless insane; how evidently their medical guardians are animated by the most generous feelings and zeal for their welfare. Thus we find them here, at Venice, encouraged to employ themselves; rewarded for their work; diverted by amusements and recreations; and instructed in religion. Their excellent and skilful physician, Dr. Pelt—to whom I owe my best thanks for much kindness—is fully alive to the deficiencies of the asylum, and anxious in every way to remedy them. He deplores its confined limits, and expresses himself decidedly against the separation of the laundry, as a place of employment for his patients, from his own jurisdiction and the confines of the asylum. But in Venice Proper—built as it is on a group of mere mud-banks, but a few feet above water-mark, and united by bridges—no spot can be found at all fitted for an asylum. Such the mainland alone can supply; yet, in spite of the multitude and the magnitude of the evils attaching to the present institution, the day is, I fear, far hence before a transfer will be made.

*The Asylum of St. Servilio,* for the male lunatics of Venice and of a considerable part of Lombardy, is, as we have already remarked, under the management of the religious brotherhood of St. Jean de Dieu. It occupies a small island in the Lagoon, about a mile from Venice, of which it commands a fine view.

The whole area of the island does not exceed four acres, and nearly one-half of it is covered by the buildings, which, although principally arranged around an inner court, as a hollow square, are also extended irregularly by additional sections. Besides a chapel, there is a good-sized church, distinguished at a distance by its two ornamental towers. The edifice, which was originally built for a convent, and is of considerable age, is of stone and brick, and of three stories. The visitor reaches the principal door in his gondola: for the walls on two sides are washed by the Lagoon, which, fortunately, has at this part a considerable current; few cases of fever are therefore met with.

The windows are everywhere guarded externally by iron bars: the present director, however, proposed to remove them from most parts of the building. The windows of single rooms, in which refractory patients sleep, are defended inside with wooden shutters, perforated by a square opening, high up, to admit light. The windows throughout are of sufficient dimensions—generally about 5ft. high and 3ft. wide—and constructed with wooden frames.

The height of the rooms on the ground floor is considerable—from 15 to 20 ft.: the largest of these rooms are subdivided by two rows of columns along their length, to support the floor above. On the first and second floors the elevation does not exceed 10ft. The floors are everywhere formed by the marble concrete.

Besides the principal court, there were two smaller ones: one set apart for the noisy and refractory patients; the other surrounded by the general offices,—the kitchen, dispensary, bakehouse, flour-store, &c. The wash-house formed a separate out-building. The kitchen was not in a good state; but it was the intention of the authorities to build another.

The visiting-room was fitted with a sort of counter extended across it, on one side of which the patients were placed during their interviews with their friends, who stood on the other. Among other necessary apartments were, a dead-house and post-mortem room, an office for the director, and a small museum.

The church used by the patients is of moderate size, ornamented with a few good paintings and with decorated altars, according to the practice of the Church of Rome. All the patients whose condition admits it attend once every Sunday, and many also on other days of the week.

The great majority of the patients sleep in dormitories, most of which contain from twenty to thirty beds, and several of them occupy the entire width of the building. Their walls are whitewashed like those of other rooms, and bare of ornament, except here and there a crucifix or the figure of a saint: at night they are faintly lighted by a lamp. Some of them,—chiefly those on the ground floor,—were less clean than could be wished, and smelt disagreeably of urine. The infirmary is large and lofty, and adjoining to it is a spacious and cheerful dormitory, set apart especially for convalescent infirmary patients.

Single rooms are placed both on the ground floor and on the upper floors, on each side a corridor, about 11ft. wide. They are about 13ft. square, and are severally furnished with a bed and a small night-commode, and in some cases with additional furniture. Small inspection doors, about 9in. square, are placed in the door of each single room; one such room, having its walls covered with a composition to give them greater softness, was used for seclusion, and no light being admitted, the patient was in complete darkness.

In a few rooms two patients were placed together, each having a separate bed. Every dormitory has a small lamp burning in it during the night.

The ordinary bedsteads and bedding resembled those in use in the female asylum. Dirty patients were provided with a wooden crib, which is filled with straw, and fitted with a wooden frame supporting a piece of canvas, not stretched out, but hung loosely, resting upon the straw, so that it may form a sort of fixed sheet. Upon this the patient lies, and is covered over by the usual coverlets. By this contrivance the advantage of the straw as a bed is secured, for the patient cannot get at it to strew it about; while at the same time he has a softer and better bed than could be afforded him if the canvas were tightly extended upon the frame, upon the plan of the stretchers so much used in this country, and which are both hard and cold. To resume the description. The bottom of the crib is perforated by a hole, through which the water escapes into a wooden drawer about 2ft. long and 1ft. wide. The straw is changed when found wet; but the fixed sheet upon it does not receive that attention which is desirable, for it frequently appeared to be allowed to dry in its place, instead of being removed and properly washed.

An attendant sleeps in each dormitory, and during the night four are employed watching and perambulating the building. The patients are attended to on going to bed; but the dirty cases are not afterwards disturbed, for the director holds it to be a very injurious plan to break the rest of the insane.

There were two bath-rooms, each containing two stone baths, sunk in the floor two-thirds of their height, and without lids; they did not appear to be much in use, and were employed only medically. Arrangements existed for affixing above them a douche-pipe. A third bath-room was occupied by a cold plunge-bath, eighteen feet by ten feet, with four feet of water, which was daily used during the continuance of warm weather. No provision for washing the hands and face was seen in any part of the building.

At the period of my visit there were three hundred and thirty patients in the institution, of whom about forty paid for their maintenance, the rest being paupers. Just as in the case of the female asylum, the pensioners lived in a separate section of the building, fared better, and had a separate day-room. The indigent patients eat together in the dining-rooms which severally belong to their separate divisions. The tables are of stained wood: they are allowed forks and spoons, but no knives; and three meals a day, in two of which meat enters as a part, except on fast days. Like the female patients, the men are dressed uniformly—during winter, in woollen jackets and trousers of a dark-brown or nearly black colour, with cloth caps; in summer, in blue linen or cotton trousers. Blucher boots are worn in winter; in summer, only a sort of slipper. It was pleasing to witness in this asylum an attempt to modify the clothing to meet the exigencies of particular cases; for not only could the boots be secured on the feet, where the tendency was to run barefoot, but a canvas dress, of waistcoat and trousers, was in use for destructive and dirty cases.

The management of the establishment is entirely in the hands of the Frères of St. John. The director, M. Portalupi, who is also the chief physician, is one of the fraternity, and has under him seven of his own order. These act as heads of departments and chief attendants, and employ a number of hired sub-attendants, who are under no religious vows. There is also a visiting physician.

The asylum was conducted on a similar system to that for the opposite sex. The value of employment was very justly appreciated, and the encouragement to work was aided by the distribution of rewards. Everything required for the use of the establishment was prepared within its walls; hence, for the first time out of England, we met with shoemakers, tailors, carpenters, smiths, bakers, &c., at work at their several trades. The washing was also done on the premises. In all, about eighty were regularly employed in mechanical work. Among the provisions for the comfort of the inmates was a library of books; and the gallery of communication between the private apartments of the managers and the workshops, was constructed as a greenhouse and filled with flowers, tended by patients.

Those not engaged in any sort of work are taken out twice a day into the garden; for, in respect of space, the male asylum enjoys a great advantage over that for the females. Although the island is so small that it cannot allow much room for garden or cultivated ground, yet it suffices to give employment to a few, and furnishes a tolerably-sized garden for exercise. This garden is planted with trees, laid out in walks, and has in its centre a high artificial mound, surmounted by a summer-house, from which a charming view is obtained over the whole vicinity, including the beautiful city of Venice itself, the islands dotting the Lagoon, and the mainland in the distance. Two other plots of land, like the garden, walled round, are set apart for cultivation—one of them as a kitchen-garden; and to render the rustic character of the establishment complete, cows are kept to supply its inhabitants with milk.

The same system of classification was in use here as in the sister asylum, and the same principles of treatment. Bleeding was very rarely resorted to; opium and morphia were occasionally used as narcotics; prolonged baths had not been tried, and the use of the douche was limited to refractory cases, as an instrument of repression. The stomach-pump was very seldom employed to feed refractory patients, their rejection of food being overcome by other means.

Mechanical coercion is considered a necessary instrument of treatment, and is effected by similar means to those enumerated in preceding pages. Still, its use was not widely extended; for out of the three hundred and thirty patients, not more than five were seen by me under restraint. It is chiefly resorted to for homicidal and suicidal cases, of which some twenty-four were reckoned to exist at the date of my visit. The contrivances in use were, the camisole, belt, handcuffs, the "muff," stiff leather gloves, and a woollen jacket made to button in front, and at the side seams, so that when buttoned upon the patient the arms were confined to the sides. In rare cases, the legs were confined by a belt of sufficient length to allow the wearer to move along. The leathern gloves were exclusively used in cases of self-abuse, and, as I understood the director, except these no instruments of restraint were employed during the night.

It should be mentioned that this establishment of St. Servilio, although chiefly limited in its purpose to the care and treatment of the insane, is, in addition, an hospital for a limited number of surgical cases, which occupy two large wards, and are under the charge of the brethren. The retention of such cases is explicable by reference to the history of the institution, which makes known to us that, prior to its adaptation for lunatics, it was an hospital chiefly for surgical patients, and that the care of the insane was only a subsequent task undertaken by the good monks.

My visit to this Asylum of St. Servilio afforded me much gratification. Its managers were evidently at work in the right direction. Its wards were generally in good order, and clean; its mechanics well occupied, and its non-industrial population well looked after and exercised; and I am persuaded, from the humane and intelligent character of the director, that he would be one of the first to abolish restraint, could he shake off the trammels of the dominant opinion of his country, and witness the non-restraint plan of treatment in practical working.

*Verona.*—This ancient Italian city, which offers so many objects of interest and attraction to the tourist, and is of so great importance to its possessors as a first-class fortress, possesses no asylum, properly so called, for the insane. The only provision made for them is such as reflects the highest discredit upon the Austrian administration of this province, and is in itself productive of misery, wretchedness, and cruelty to the unfortunate patients.

The accommodation provided is in connexion with the large general hospital—an irregular group of buildings, some of considerable age, and others of modern construction. The lunatic department is very subordinate, and greatly neglected: it occupies several small buildings and wings, very ill adapted to their purpose—some of them very ill built, and all of them wretchedly kept—assigned to the helpless insane as the least eligible and useful for any other purpose, and because some sort of habitation must perforce be found for them. Both sexes are received, and the credit must be given to its managers that they are placed in completely distinct buildings. The women occupy three small, low-vaulted dormitories on the basement, with stone floors, badly lighted and ventilated, and altogether very dull and miserable.

A few of the male patients, who are very quiet and tractable, live separate from their refractory companions, but are, nevertheless, very indifferently off for accommodation. They have a small garden to themselves for exercise. The refractory, who constitute the majority of the male patients, are lodged in a small, detached, low building of one story, with barred windows and a stone floor, so laid as to slope from each side towards a grooved drain running from one end to the other. The whole aspect of this apartment was rather that of a stable than of a dormitory for the residence of human beings—indeed, many English stables are far more warm, sweet, and comfortable. The occupants of this room were disposed in beds arranged in a row on each side, and, so far as I could learn, were very rarely permitted to leave their beds. No effectual means of enforcing this condition of repose were omitted; the hands and feet were made fast by leathern straps to the bedstead, and, if required, a belt across the body could be added. The bedsteads were of wood, very thick and heavy, and constructed in a crib-like fashion, filled with straw, over which a very coarse brown linen sheet or piece of canvas was laid, to serve as an under-sheet for the patient. Another such sheet covered him, with the addition of a very coarse woollen coverlet. Being unable to help themselves, the urine was always necessarily passed under them, and after percolating through the straw, diffused itself over the stone floor, until it reached the central gutter, whence it could flow along to one end of the building and escape. The mode of distributing food to these unfortunates was on a par with the rest of the treatment. The soup—their only diet, except bread—was brought to them in pails, out of which a basin was filled for each. As they were helpless by restraint, the services of their attendants were required to feed them. We must add to the above account, that the heads were generally shaved, and that the phlebotomist had constant employment to keep under (?) the cerebral excitement, indicated by the perpetual noise and restlessness of the refractory inmates.

To render to every one his due, we must state that the soup and bread appeared of good quality, and well adapted for nourishment—better, indeed, than unfortunately falls to the lot of a large part of the poor of Lombardy.

The male attendants belonged to a religious order—as I understood, of St. Euphemia. There was no special physician for this section, its duties falling casually upon the physicians of the general hospital—particularly upon the medical director, as superintendent of the entire establishment. The offices, kitchen, laundry, dispensary, &c., were common to the whole institution.

When I made my visit, there were forty insane patients in this section of the Verona Hospital; of these, eight were pensioners, permanently resident. Happily their condition was tolerably satisfactory; they occupied a distinct building, having a small garden, and each one had his own room, sufficiently furnished and comfortable. When quiet, considerable liberty was accorded them to walk about in the grounds of the hospital.

The pauper patients are, unless there is speedy prospect of their recovery, retained at Verona for only two months, at the end of which time they are sent to the asylum at Venice, with small prospects, we apprehend, of deriving much benefit when they get there, the preliminary treatment they have undergone at Verona being taken into consideration.

Our readers will peruse with pain and astonishment this account of the treatment the insane receive in a large city of the Continent in these latter days of enlightenment and charity; but we greatly fear that, if the condition of the insane in many of the large Lombard cities (in the general hospitals of which they occupy an inconsiderable and neglected section) were inquired into, Verona would not be found exceptional in its mode of treating them. Lombardy, alas! is not only now a very impoverished country, but is held in rigorous subjection to a foreign race by military force, to the support of which, as well as of the civil government, it is made heavily to pay; and, as a consequence, no funds are to be found for such purposes as the building and endowment of proper lunatic asylums.

*Brescia.*—Within the walls of this city is a nearly new asylum, which constitutes an appendage of the general hospital, and to which it is united by corridors. As in similar instances, the general offices are common.

The building forms two hollow squares, one assigned to each sex, with an intervening garden of about half an acre in extent. It has only two floors—a basement or ground, and a first floor: around each is a covered corridor. That on the first floor is nearly ten feet wide, and on its open side, looking into the central court, forms a series of circular-headed spaces with intervening square columns, the open spaces being filled in with upright iron rods, crossed by others at intervals of about two feet. This corridor would have a very good effect were it not for its barricaded spaces, which give it a cage-like appearance. The ground floor is occupied by the day and dining-rooms, by some single rooms, and by the bath-room. The concrete of the floors of these rooms is laid immediately upon the earth, and, in consequence, is frequently moist from the transudation of moisture. One of the day-rooms on the women's side was small, badly lighted by one window, and so constructed that one half was at right angles to the other.

On the first floor were two dormitories, called infirmaries, both of which were unoccupied on the male, and one on the female side. The larger one on each side would contain from twenty-four to thirty beds; the smaller was about half the size. My visit was made in March; later in the spring, I was assured, these vacant rooms would be filled with cases of pellagra. The elevation of these dormitories was good, viz., about fourteen feet; but they were rendered dull and dreary by the half-circular small windows being placed seven feet above the floor. Except the portion occupied by the infirmary dormitories, the rest of the first floor was constructed with a corridor, having a row of single rooms on each side, but not terminated by an end window. These rooms were about twelve feet by ten feet, and contained each a bed, a fixed seat at one corner, and generally a recess in the thickness of the wall, to serve as a receptacle for the clothes of the occupant.

The window of each single room was rather high up, barred on the outside, and its wooden frame covered, in place of glass, with canvas sufficiently thin to admit light. This mode of filling a window-frame has certainly the merit of economy, and may not be uncomfortable on a warm summer's night in this part of the world; but we much pity the unfortunate patients who have to pass a long winter's night in rooms so imperfectly shut from the outer air. In the infirmaries, indeed, the windows were glazed. The doors of the single rooms opened outwards: the floors were of concrete or stone.

Some of the stairs were winding, and, to obviate danger, their "well" was covered at the landings by wirework. In this, as in the other Italian asylums described, little attention was bestowed upon warming the rooms in cold weather; the discussions of systems of warming and ventilating asylums in this more northern clime have not, it would appear, aroused the attention of Italian physicians and architects. Certainly they have much less need to make provision against cold; yet, notwithstanding, a winter in northern Italy is not to be braved without fires, and much suffering must be entailed upon the inmates of public institutions by the prevailing absence of any attempts at systematic warming. Here, in Brescia, an Italian stove, placed in the centre of one or two of the day-rooms, surrounded by stout guards, was all the means provided.

A small chapel, calculated to accommodate about fifty persons, had been built for the patients; it presented nothing remarkable to note.

I found many patients in bed, not apparently labouring under any bodily sickness demanding repose, but placed there apparently as a means of seclusion and confinement. In the case of the men, I noticed that all, or nearly so, were under restraint, attached by a foot or an arm to the bedstead. Indeed, restraint was used in this asylum in an irregular, loose, and most reprehensible manner. No employments and no amusements for the patients were attempted; the construction and very limited space of the building admitted of no proper classification, and, what was worse, none appeared thought of; hence confusion, disorder, noise, and misery reigned supreme, and the only check the managers could devise was that furnished by mechanical restraint. This appeared not only in the poor prisoners in their rooms, condemned to seclusion and the tedium of bed, left to cherish their disordered fancies and to sink deeper into despair, but in many more besides, wearing camisoles, handcuffs, and here and there one with hobbles on the feet.

To the same want of management, to the same neglect, were due the frequently disorderly or ragged clothes, the naked feet, and the dirty habits of many of the inmates.

The bedsteads for refractory cases were well supplied with rings at the head, foot, and sides, for the passage of straps for restraint: the dirty slept upon straw, through which the urine percolated on to the floor, no attempt being made to collect it. No special provision for epileptics and paralytics was thought of; but they were treated as ordinary cases, and slept on straw as dirty cases, or had beds like the clean,—consisting of a paillasse, a flock bed, a pair of sheets, a woollen, and over all, a striped cotton coverlet. The bedsteads, except those for foul cases, were of iron.

Rather over two hundred patients were detained in this asylum at the period of my visit, and, as elsewhere, formed two classes—pensioners and paupers—the former, however, in very small proportion. For the number resident, the accommodation was much too small.

Although a section of the general hospital, it had its special physician, who visited once or twice daily. An interne on the male side, and a sort of matron on the female, each with the title of inspector, are in constant residence within the building.

Not having had the advantage of being accompanied over the institution by the physician himself, but by a colleague attached to the general hospital, there are many matters touching the internal regulation of the asylum, and the treatment pursued, which I could not ascertain. Respecting the medical treatment, I learnt that bleeding, both general and topical, was very much resorted to, and that opiates were very seldom employed. Pellagra affected at least one-half of the population of the asylum.

The garden between the two divisions was common to both sexes; but they were taken for exercise in it at different hours of the day. The view from the garden, and indeed from the asylum itself, is very limited, by the proximity of the ramparts of the town, which are considerably raised above the level of the land inside them.

Unlike several of the Italian asylums, that of Brescia had the advantage of being specially built for its purpose—an advantage, however, almost completely negatived by the very faulty plan of its construction, and, for every good result, entirely sacrificed by bad management. All the modern improvements and ameliorations in the condition of the insane, and all the teachings of modern pathology respecting their treatment, appear either unknown or uncared-for by the authorities at Brescia. There is great need for some asylum reformer to visit the institutions of Lombardy, and to teach the doctrines of Pinel, Esquirol, Conolly, and of the other noble men who have laboured in the cause of the insane.

*Aversa.*—The town of Aversa is situated about midway between Naples and Capua. Its name is well known to psychological physicians, because the principal public asylums of the kingdom of Naples, which are in its immediate neighbourhood, gained for themselves much credit by being among the first to employ their lunatic patients in various kinds of work. However, at the present period they would occupy but a low place in the scale of merit among the asylums in Europe; for, although the advantages of employment are still duly appreciated, yet they have not in other respects followed the march of progress in the moral management of the insane, which has so rapidly advanced in this country and elsewhere.

The two sexes are accommodated in distinct buildings: that for the women is in the town of Aversa; the one for males is some distance from the town, on the high road towards Capua. A third institution exists in the vicinity, for epileptic and imbecile cases, under the direction of a religious fraternity. The asylum for females, in Aversa, I did not see, but heard that it was a very indifferent and unsuitable building. The one for the men is considered the best; unfortunately, my examination was imperfect and hasty, as I found no medical officer in the establishment, and had only one of the attendants to act as my conductor through it, and had no opportunity of repeating my visit.

The situation of the asylum is very good; the surrounding country of great beauty, and well cultivated; but, owing to walls and out-buildings, the beauty of the scenery is hidden from the inmates, except when in their sleeping-rooms upstairs. The building is partly erected in the form of a hollow square, but is rendered irregular by other portions added from time to time. The principal edifice is of three stories in height, whilst other parts have only two floors. Except three moderately-sized airing-courts, no other ground is attached to the institution. On entering the building, the visitor first reaches an enclosed court, surrounded by the general offices and work-rooms, which occupy the ground floor, and have dormitories above them. This court is surrounded by a covered corridor or arcade.

Owing, doubtless, to progressive alterations and additions, there is an irregularity in the interior, some wards being on a different level to others, properly speaking, on the same floor, and therefore reached only by mounting or descending a few stairs; moreover, from the like cause, the elevation of the different rooms varies considerably, and in some is deficient. Their floors are paved with tiles, their walls whitewashed and bare, and the windows barred externally. Although little objection could be taken to the degree of cleanliness generally presented by the asylum, yet, taken as a whole, its interior aspect was dull and heavy, and did not realize an Englishman's idea of comfort.

I saw no single rooms, although several small ones existed in which two patients were placed together. The dormitories differed in size—the largest contained from twenty to thirty beds: in each of them an attendant sleeps, in a small apartment partitioned off, but so constructed as to command a view of the entire room. A light is burnt in every dormitory during the night. To meet the wants of the patients when in their sleeping-rooms, a large pail or tub is attached by a chain to the wall at one end of the room, for chamber utensils are not allowed. The upper floors are, at most parts, built with a central corridor, having rooms opening from it on each side.

The bedsteads consist simply of some flat boards, resting on iron trestles at the head and feet: on these are placed a palliasse, a flock bed with sheets, a woollen and a cotton coverlet. Dirty patients were allowed a palliasse only, and no bed or sheets. For the particular forms of insanity complicated by paralysis and epilepsy, no specially constructed beds were supplied. During the day the bedding was rolled up towards the head of the bedstead, after the fashion seen in barracks and in a few of our own asylums.

The patients were nearly three hundred in number, and of two classes—pensioners and paupers—the former in comparatively very small proportion. The attendants seemed tolerably numerous; and owing to the greater part of the inmates being occupied at work, there was an air of activity, quiet, and content, not witnessed in those asylums where employment is omitted or neglected. Some of the inmates work as tailors, others as shoemakers, &c.; but the majority are occupied in the large weaving-room, at hand-looms, making cotton and linen cloth for the use of the institution, and as an article of sale. The room for the weavers was of large size: the duration and the amount of work exacted from the patients seemed too great, whilst means of amusement and opportunities for recreation were too much neglected. Of the several modes of employment in which the insane are engaged, that of weaving appears to me one of the worst adapted to their condition, by reason of the confinement it involves, the monotony of the task, and the imperfect character of the exercise it calls upon them to take. The work certainly is anything but exhilarating; the atmosphere of a weaving-room becomes dusty and close, and the clatter of the machines dreary by its sameness, and musical to no other ears except those of the master, who reaps the fruits of the work. In short, weaving is a description of labour inimical to mental discipline and development, and opposed to the physical well-being of the body.

I should have rejoiced to have seen the inmates of Aversa engaged in gardening and tillage; but this, the most healthy and cheerful employment, is neglected; in fact, the asylum possesses no land for cultivation. The only outdoor provision for the patients is furnished by the three small exercising courts, which are of themselves sufficiently agreeable, although inadequate to the wants of the asylum, and dull by not affording any view beyond their restricted boundaries. They were, however, pleasantly planted with trees and flowers, and gave occupation to two or three inmates.

Restraint, I was told, was little resorted to; but neither of this matter, nor of the general moral and medical treatment, have I, unfortunately, any details. The general impression I formed respecting this asylum of Aversa was, that it was far behind, in construction and management, the asylums of this country, and many of those of France and Germany. The irregularity of the interior, its bare walls, barred windows, the absence of the means of amusement, and the seeming pursuit of employment as a source of profit, imparted to the whole institution a dull, heavy, comfortless aspect, and, owing to its limited outdoor space, the air of a place of confinement.

I have met with the following statistics of the asylum under consideration, in connexion with that for the females:—Between 1813 and 1841 inclusive, there were admitted 4165 men and 1741 women; a total of 5906. Of these, 1535 males and 600 females were discharged cured; 612 men and 148 women, uncured or as found not insane; whilst 1486 and 758 of each sex severally died; a total of 2244. Taking these figures, the per centage of cures on the number of admissions appears to be 36·1; and that of deaths, 37·9. The statistics of 1841 were as follows:—Remaining in the establishments, 532 males and 146 females; total, 678. Admitted of each sex severally, 186 and 90—276 in all. Discharged cured, respectively, 63 and 18; uncured and not insane, 39 and 13—a total of 133 discharged; deaths, 84 men and 70 women; in all, 154. Hence the per centage of cures was 29·3 on the admissions alone, and when calculated on the total of admissions and the previous number under treatment, only 8·4; whilst the proportion of deaths to the admissions was as high as 55·7; or to the admissions, plus existing cases, or the total population of the year, 16·1. Between 1846 and 1848, inclusive, the figures were:—Admissions, 599 males, 248 females; cured, 201 and 75; uncured and not insane, 143 and 55; died, 267 and 93. Thus the cures to the admissions were 32·5 per cent., and the deaths 42·5.

These statistics prominently show, among other facts, how much larger is the number of male lunatics admitted than of females—viz., two-thirds more. Another striking fact elucidated is the very high rate of mortality, which exceeds that of most asylums of Europe, if we except the Parisian, in which general paralysis makes such terrible havoc.

---

### Art. IV.—THE CONDITION OF THE INSANE.

